# Functional characterization of fungal endophytes with antagonistic and plant growth-promoting activities in maize

**DOI:** 10.1038/s41598-026-60768-4

**Published:** 2026-07-11

**Authors:** Purusottam Majhi, Jharana Pradhan, Umakant Pradhan, Sandeep Kaushik, A. K. Shukla

**Affiliations:** 1 Microbiology Laboratory, Department of Botany, Indira Gandhi National Tribal University, Amarkantak, 484887 Madhya Pradesh India; 2 Department of Environmental Science, Indira Gandhi National Tribal University, Amarkantak, 484887 Madhya Pradesh India

**Keywords:** Endophytic fungi, Mycorrhiza, Antifungal activity, Plant growth promotion, Metabolite production, Phytopathogens, Photosynthetic pigment, Biotechnology, Microbiology, Plant sciences

## Abstract

Fungal endophytes play a crucial role in plant-microbe interactions by asymptomatically colonizing host tissues, enhancing plant growth, and providing defense. In the present study, three distinct endophytic fungi, namely *Fusarium oxysporum* CJR-1 (GenBank Accession No. PZ477045), *Penicillium* sp. CJR-2 (GenBank Accession No. PZ477429) and *Aspergillus cf. terreus* CJR-4 (GenBank Accession No. PZ477430) were isolated from *Crotalaria juncea* L. plants and evaluated for antagonistic activity against phytopathogenic fungi and growth promotion in maize. Among the isolates, CJR-4 showed potent antagonistic activity against both tested phytopathogens, inhibiting the mycelial growth of *Fusarium oxysporum* (ITCC Accession No. 8111) and *Alternaria alternata* (ITCC Accession No. 1434) by 72.41 ± 5.02% and 68.31 ± 1.53%. On the other hand, isolate CJR-1 showed the highest mycelial inhibition of 74.60 ± 2.06% against *A. alternata*. Ethyl acetate crude metabolites of CJR-4 exhibited potent antifungal activity, with the highest zone of inhibition observed against *A. alternata* (21.66 ± 1.52 mm), followed by *F. oxysporum* (15.33 ± 1.15 mm) at 150 µL of tested concentration. GC-MS analysis of the ethyl acetate extract of CJR-4 revealed numerous bioactive metabolites, including diphenyl sulphone and 2-nonen-1-ol, which may contribute to its antifungal activity. In addition, 1-methylene-1H-indene and ethanone, 2-(formyloxy)-1-phenyl-, may be associated with antioxidant activity, as evidenced by potent DPPH (80.09 ± 0.11%) and ABTS (92.66 ± 0.17%) scavenging activities. Evaluation of PGP traits showed that CJR-1 produced the highest levels of indole-3-acetic acid (IAA) as 305.69 ± 0.41 µg mL⁻^1^ and ammonia 85.44 ± 0.63 µg mL⁻^1^, while CJR-2 exhibited the highest phosphate solubilization index (PSI) of 3.09 ± 0.09. Pot experiments conducted under natural light conditions for 28 days demonstrated significantly enhanced plant growth parameters, biomass accumulation, and photosynthetic pigment levels in maize plants inoculated with the CJR-4 isolate. Overall, the findings highlight that endophytic fungi exhibit multifunctional roles and show promise as microbial inoculants for sustainable crop improvement, with CJR-4 emerging as the promising candidate for further development. To the best of our knowledge, this study is among the first to report the isolation and functional characterization of endophytic fungi from *Crotalaria juncea* L. plant.

**Supplementary Information:**

The online version contains supplementary material available at 10.1038/s41598-026-60768-4.

## Introduction

Endophytic fungi are important components of plant-associated microbial communities that colonize inter-or intracellular plant tissues without causing apparent harm and often establish symbiotic interactions with their hosts^1^. These symbiotic microbes have attracted considerable attention for their ability to enhance plant growth, improve nutrient acquisition, and protect plants from both biotic and abiotic stresses. In modern agriculture, the intensive use of agrochemicals has been associated with the deterioration of soil health and the disruption of beneficial microbial communities, leading to soil infertility and long-term sustainability concerns. To mitigate these issues, plant growth-promoting microbes, or biofertilizer, are gaining popularity as an alternative to agrochemicals. Inoculation with biofertilizers containing beneficial fungi or bacteria results in colonization of the host plant. It promotes plant growth and crop production through mineral solubilization and the production of phytohormones and secondary metabolites that suppress phytopathogens^2,3^.

Fungal endophytes mitigate biotic and abiotic stress by direct and indirect mechanisms and facilitate plant growth. The direct mechanisms include stimulating the production of bioactive substances, such as phytohormones, ammonia, siderophores, and the solubilization of phosphorus and potassium^4,5^. In addition, indirect mechanisms include the suppression of phytopathogens and the induction of systemic resistance in the host plant, facilitated by the production of antibacterial, antifungal, and antioxidant compounds, as well as antagonistic interactions^6^. Endophytic fungi are known to produce numerous phytohormones, such as Indole-3-acetic acid (IAA), gibberellin (GA), salicylic acid (SA), and cytokinin (Cks), which contribute to plant growth and development^7,8^. Even at low concentrations, the most common phytohormone, IAA, facilitates elongation of primary roots and increases lateral root and root hair development^9^. Additionally, they play a crucial role in mineral solubilization and the acquisition of nutrients such as nitrogen, phosphorus, and other micronutrients, thereby enriching soil nutrient availability and reducing dependency on chemical fertilizers^10^. Moreover, they evade plant stress by producing defense signaling molecules, such as salicylic acid, jasmonic acid, and abscisic acid, as well as by improving nutrient uptake and boosting biomass, making them a key player in sustainable agriculture^11^. In addition, endophytic fungi isolated from various plants, such as *Helianthus tuberosus*, *Chromolaena odorata*, *Zingiber officinale*, *Stemona sessilifolia*, *Ligusticum chuanxiong*, and *Tetraena qatarensis*, have been reported to produce diverse plant growth-promoting metabolites and enzymes, including hydrogen cyanide (HCN), IAA, siderophores, and extracellular enzymes^8,12,13^.

Several studies have demonstrated that fungal endophytes belong to genera such as *Fusarium* sp. *Aspergillus* sp. *Penicillium* sp. and *Trichoderma* sp. significantly enhance plant growth and crop productivity. For instance, *Fusarium oxysporum* FO12 inoculation in soybean and wheat has been reported to increase plant height and biomass, along with enhanced uptake of macro- and micronutrients compared with untreated plants^14^. Similarly, *Aspergillus terreus* isolated from *Catharanthus roseus* enhanced *Vigna radiata* growth through IAA production, phosphate solubilization, and ACC deaminase activity^15^. Likewise, *Penicillium bilaii* has been reported to improve phosphorus availability and root development^16^. In addition, treatment with *Trichoderma* strains in tomato seeds significantly enhanced plant height, biomass, leaf area, and chlorophyll content^17^. Similarly, inoculation of *Aspergillus niger* with sunflower and soybean plant has been reported to significantly enhance plant height, biomass, and chlorophyll content^18^. These findings highlight the multifunctional roles of fungal endophytes in plant growth promotion and their potential application as bioinoculants.

*Crotalaria juncea* L., also known as brown hemp, Indian hemp, Madras hemp, or Sunn hemp, is a shrub belonging to the Fabaceae legume family and is considered to have originated in India^19^. Due to its high nitrogen content, it is used as a green manure, improving soil quality and crop sustainability by minimizing external nitrogen inputs, N_2_O emissions, and nutrient leaching^20^. Additionally, recent studies have shown that *Crotalaria* improves soil microbial communities, promotes plant growth, and controls phytopathogens^21^. In particular, *Crotalaria juncea* L. is reported to be a pyrrolizidine alkaloid plant that reduces phytopathogens such as nematodes and pests^22,23^. To the best of our knowledge, no earlier data on the isolation and functional characterization of fungal endophytes associated with this plant exist, despite its agronomic significance.

Therefore, the present study aimed to isolate fungal endophytes from the roots of *Crotalaria juncea* L., and investigate their antagonistic activity against phytopathogenic fungi, along with their plant growth-promoting potential. Selected isolates were assessed for key PGP traits, including IAA production, siderophore production, ammonia production, and phosphate solubilization, and their effects on maize growth were further evaluated under pot conditions. This study aims to isolate and identify promising multifunctional endophytic fungi with potential as bioinoculants for sustainable, eco-friendly agriculture.

## Materials and methods

### Materials used

All chemicals and media used throughout this study were procured from Himedia, CDH, Qualigens, and Rankem, India. Mili-Q grade water was used to conduct the experiments in the study. HiPurA Fungal DNA Purification Kit, 2X PCR TaqMixture, 1kb DNA Ladder, Hi-SYBr Safe Gel Stain were procured from Himedia Laboratories Pvt. Ltd., and internal transcribed spacer (ITS) primers for molecular identification of endophytic fungi were obtained from Eurofins Genomics India Pvt. Ltd.

### Plant sampling and study area

The plant *Crotalaria juncea* L. was collected from the campus of IGNTU-Amarkantak, M.P., India, a transition zone of the Achanakmar-Amarkantak Biosphere Reserve. The fresh, healthy plant samples were packed in sterile zipper bags and brought to the laboratory. Dr. Ravinder Shukla (Associate Professor), Department of Botany, IGNTU-Amarkantak, identified the plant and the voucher specimen was deposited in the departmental herbarium (Accession no: IGNTU/DoB/2023/Herb-Feb/Cj-02).

### Source of phytopathogenic fungi and maize seeds

The phytopathogenic fungal strains used in this study were procured from the Indian Type Culture Collection (ITCC), New Delhi, India with accession numbers ITCC 8111 (*Fusarium oxysporum*) and ITCC 1434 (*Alternaria alternata*). Maize seeds used in the pot experiment were collected from local farmers of village Lalpur, Anuppur District, Madhya Pradesh, India. The seeds were healthy and free from visible disease symptoms.

### Fungal isolation and cultivation

Endophytic fungi were isolated from the roots of the collected plants. Surface sterilization of plant samples and isolation of endophytic fungi were carried out following the protocol of Tan et al.^24^ with slight modifications. Plant roots were detached from the plant and thoroughly washed 2–3 times in running tap water. All the roots were transferred into a beaker containing 70% ethanol for one minute, then immersed in 2% sodium hypochlorite solution for 5 minutes, followed by successive rinses with sterile distilled water, and then dried on sterile filter paper inside a laminar airflow. Sterilized plant roots were cut into small fragments and aseptically placed on the potato dextrose agar (PDA) supplemented with streptomycin sulphate (30 mg/L). To ensure the sterilization of plant samples, 100 μL of final rinse water was pipetted separately into a petri plate containing PDA medium. All the petri plates were then wrapped with parafilm and incubated at 28 °C for 7 to 10 days in a BOD incubator. Hyphae from the emerging colonies were again subcultured in PDA media for 5 to 7 days to obtain a pure culture.

### Morphological identification

Identification of the isolated endophytic fungi was carried out based on their morphological and microscopic structures following the fungal standard manuals^25,26^. Isolated endophytic fungi were cultured on PDA and observed their colony morphology like, growth rate, growth pattern, texture, pigmentation, and reverse plate colouration. Slide of the isolated endophytic fungi were made using lactophenol cotton blue and observed using compound microscope.

### DNA extraction, PCR amplification, Sanger sequencing, and identification

Prior to DNA isolation, the endophytic fungi were grown on PDA medium at 28 °C for 10 days. Then the mycelia were scraped with a sterile needle, placed into a 2 mL Eppendorf tube, frozen in liquid nitrogen, and thoroughly broken using a micro pestle. The fungal DNA was isolated using the HiPurA Fungal DNA Purification kit (Himedia HiGEnoMB, Mumbai, India) according to the manufacturer’s protocol. Universal primer ITS1 (forward): 5′-TCCGTAGGTGAACCTGCGG-3 and ITS4 (reverse): 5′-TCCTCCGCTTATTGATATGC-3′ were used to amplify the ribosomal internal transcribed spacer regions for molecular phylogenetic analysis^27^. The PCR mixture of 25 µL contained 12.5 µL of 2X PCR TaqMixture (Himedia, HiGEnoMB), 1 µL of each primer at 10 µM, 2 µL of template DNA, and 8.5 µL of molecular grade water. Amplification was carried out by following the company protocol with slight modification using a thermal cycler (MiniAmp Plus) as initial denaturation at 95 °C for 2 min, followed by 30 cycles at 94 °C for 1 min, 56 °C for 30 s, 72 °C for 53 sec, and then a final extension step at 72 °C for 5 min. Furthermore, the PCR products (2 µL) were electrophoretically analyzed in 1% (w/v) agarose gels stained with Hi-SYBR Safe Gel Stain, and the gels were subsequently visualized and compared with a 1kb DNA ladder using a gel documentation unit (GelLITE, Cleaver Scientific). The Sanger method was used to sequence purified PCR products at BioEdge Solutions in Bangalore, India. The endophytic fungal isolates were identified using the Nucleotide Basic Logical Alignment Search Tool (BLAST) in the NCBI server database (http://www.ncbi.nlm.nih.gov). The obtained ITS sequences were subsequently deposited in the NCBI GenBank for accession numbers. Multiple sequence alignment was done by ClustalW, and the maximum-likelihood phylogenetic tree was constructed using MEGA 6 software, with the Tamura-Nei model by bootstrap analysis (replicates of 1000).

### Antagonistic effect of endophytic fungal isolates against fungal pathogens

A dual culture antagonistic assay of isolated endophytic fungi was performed against fungal pathogens, *F. oxysporum*, and *A. alternata* following the method of Trejo-Estrada et al.^28^. A 5 mm disc of a 7-day-old endophytic fungal culture was placed 2 cm away from the periphery of the petri plate containing PDA medium, and another 5 mm disc of the 7-day-old challenge pathogen was placed in the same petri plate opposite to the endophyte in the same manner. Monoculture plates of pathogens served as a control. The plates of three replications of each treatment were then incubated at 28 °C in a BOD incubator for seven days. Observations of growth were made, and colony inhibition percentage was calculated using the formula:

$$\mathrm{I}\mathrm{n}\mathrm{h}\mathrm{i}\mathrm{b}\mathrm{i}\mathrm{t}\mathrm{i}\mathrm{o}\mathrm{n} \:\:\mathrm{P}\mathrm{e}\mathrm{r}\mathrm{c}\mathrm{e}\mathrm{n}\mathrm{t}\mathrm{a}\mathrm{g}\mathrm{e} = [(\mathrm{C} - \mathrm{T})/\mathrm{C}] \times 100$$

C – Radial growth of pathogens in control; T – Radial growth of pathogens in treatment plate.

### Preparation of fungal crude extract

Prior to crude extract preparation, three selected endophytes were precultured on PDA medium for 7 days. Then 4–5 plugs (0.5 cm) of each fungus were transferred into 500 mL of sterilized potato dextrose broth (PDB) and incubated at 28 °C for 15 days in a rotary shaker incubator at 140 rpm. The fungal broth was filtered through Whatman No. 1 filter paper to separate the fungal hyphal mat. The filtrate obtained was extracted thrice with equal volume of ethyl acetate and evaporated at 40 °C using a vacuum rotary evaporator (BIOGEN SCIENTIFIC: BGS-173) to yield the crude metabolites. Dried crude extract was stored at 4 °C for further study.

### Antifungal activity of ethyl acetate crude metabolites

Based on the dual culture test, crude metabolites of *A. cf. terreus* CJR-4 were tested for antifungal activity against phytopathogenic fungi *F. oxysporum* and *A. alternata*. The agar well diffusion method was employed according to the National Committee for Clinical Laboratory Standards (NCCLS) guidelines^29^. The test pathogens were initially grown on PDA medium for 3–5 days at 28 °C. Fungal spore suspensions were prepared in phosphate buffer (pH 7.0) and adjusted to 1 × 10^7^ spores/mL after using a haemocytometer. Subsequently, one millilitre of the adjusted spore suspension was pipetted and uniformly spread on the PDA plates using a sterile L- spreader. Four wells (5 mm) were made in each plate using a sterile cork borer. A stock solution of fungal crude metabolites was prepared in DMSO at 100 mg/mL, and 50, 100, and 150 µL were loaded into separate wells. Nystatin (NS 100) was used as a positive control, and DMSO as a negative control. The experiment was conducted in triplicate. To facilitate the better diffusion of crude metabolites, plates were initially kept at 4 °C for 2–3 h and subsequently incubated at 28 °C for 3–5 days in a BOD incubator. After incubation, the diameter of the inhibition zone was measured and recorded.

### GC-MS analysis of fungal crude extract

The GC-MS analysis of the ethyl acetate extract of the fungal isolate CJR-4 was performed using a Thermo Trace 1600 GC coupled to a Thermo ISQ 7610 single-quadrupole mass spectrometer with an ionization energy of 70 eV. High-purity helium gas was used as the carrier gas at a flow rate of 1.2 mL/min. The ion source and interface temperatures were set to 240 °C and 250 °C, respectively. The injector temperature was maintained at 250 °C, employing a split ratio of 1:10 through split-splitless injection of a 1 μL sample solution. The column TGS5-MS (length 30 m × 0.320 × 0.25 mm) was used. Initially, the oven temperature was set at 80 °C and gradually raised to 280 °C, with a 2-minute hold at 280 °C. The individual compounds were analyzed in full-scan mode (m/z 30–550). The compounds were identified by comparing their mass spectra with those available in the National Institute of Standards and Technology (NIST) mass spectral library. The best-matched compounds were selected based on their Similarity Index (SI) and Reverse Similarity Index (RSI) values.

### Evaluation of plant growth-promoting traits of isolated endophytic fungi

The ability of fungal endophytes to produce substances related with plant growth-promoting capabilities were determined as follows:

### Analysis of indole-3-acetic acid production (IAA)

The ability of fungal endophyte to produce IAA was determined by following the method of Fauda et al.^30^, in which isolated endophytic fungi were inoculated into Czapek Dox (CD) broth media. One disc (5 mm diameter) of each isolate was transferred to 20 mL of sterile CD liquid medium containing different concentrations of L-tryptophan (1, 2, and 5 mg/mL) or without tryptophan to act as a control and incubated at 28 °C for 10 days in a shaking incubator. Further, 5 mL of each culture was collected and centrifuged at 6000 rpm for 30 minutes to separate the mycelial mat. Then 1 mL of the supernatant was mixed with 1 drop of orthophosphoric acid and 2 mL of Salkowski’s reagent (300ml concentrated Sulphuric acid; 500 ml distilled water; 15 ml 0.5 M FeCl_3_), followed by dark incubation for 30 minutes. Orthophosphoric acid was used to maintained the acidic conditions, which enables reaction of IAA with ferric ions, resulting in the development of pink-reddish coloured complex for IAA estimation. To quantify the IAA production, optical density was measured at 530 nm using a spectrophotometer. The amount of IAA production was estimated by using the standard IAA graph. The experiment was conducted in triplicate.

### Phosphate solubilization

The ability of fungal endophytes to solubilize inorganic phosphate was evaluated by following the method of Jasim et al.^31^. Pikovskaya’s medium (0.5 g yeast extract powder, 10.0 g dextrose, 5.0 g Ca_3_(PO_4_)_2_, 0.5 g (NH_4_)_2_SO_4_, 0.2 g KCl, 0.1 g MgSO_4_ × H_2_O, 0.0001 g MnSO_4_ × H_2_O, 0.0001 g FeSO_4_ × 7H_2_O, 20.0 g agar, dissolved in 1.0 L d. H_2_O, pH 6.8) was used to evaluate phosphate solubilization capability of endophytic fungal isolates. Petri plates were incubated at 28 °C for 3–5 days in a BOD incubator. The experiment was conducted in triplicate. The diameter of the clear zones around the fungal disc indicated phosphate solubilization and was used to qualitatively determine the phosphate solubilization index (PSI). PSI was calculated using the following formula of Paul and Sinha^32^:

$$\mathrm{P}\mathrm{S}\mathrm{I} = (\mathrm{c}\mathrm{o}\mathrm{l}\mathrm{o}\mathrm{n}\mathrm{y}\:\: \mathrm{d}\mathrm{i}\mathrm{a}\mathrm{m}\mathrm{e}\mathrm{t}\mathrm{e}\mathrm{r} + \mathrm{c}\mathrm{l}\mathrm{e}\mathrm{a}\mathrm{r}\:\: \mathrm{h}\mathrm{a}\mathrm{l}\mathrm{o}\:\: \mathrm{z}\mathrm{o}\mathrm{n}\mathrm{e} \:\:\mathrm{d}\mathrm{i}\mathrm{a}\mathrm{m}\mathrm{e}\mathrm{t}\mathrm{e}\mathrm{r}) / (\mathrm{c}\mathrm{o}\mathrm{l}\mathrm{o}\mathrm{n}\mathrm{y}\:\: \mathrm{d}\mathrm{i}\mathrm{a}\mathrm{m}\mathrm{e}\mathrm{t}\mathrm{e}\mathrm{r})$$

### Ammonia production

The ammonia production capabilities of fungal endophytes were evaluated using the methods of Mahfooz et al.^33^ with minor modifications. Endophytes were inoculated in peptone water (peptone 10 g; NaCl 5 g; 1.0 L dH_2_O) and incubated for 7 days in a shaker incubator at 140 rpm. Broth without fungal inoculum acts as a control. Briefly, broth was centrifuged at 5000 rpm for 10 min, and the supernatant was collected. Then, 1 mL of collected supernatant was mixed with 4 mL of Nessler’s reagent (K_2_HgI_4_ and NaOH or KOH), and the change of colour to yellow-brown or brown indicates the positive result of ammonia production. The intensity of colour showed the degree of ammonia production by fungal isolates. Absorbance of the samples was taken at 530nm using a spectrophotometer. The standard curve of ammonium sulphate solution ((NH_4_)_2_SO_4_) was produced for the quantification of ammonia in the fungal samples^34^.

### Siderophore test

The universal CAS assay was employed to test the fungal isolates for siderophore production. The CAS reagent was prepared by dissolving 121 mg of CAS in 100 mL of distilled water, followed by the addition of 20 mL of a 1 mM ferric chloride (FeCl_3_ ⋅ 6H₂O) solution prepared in 10 mM hydrochloric acid. Under constant stirring, this solution was added to 20 mL of a hexadecyltrimethylammonium bromide (HDTMA) solution (36.45 g of HDTMA in 20 mL). The prepared CAS-HDTMA reagent was sterilized before the test^35^. For the detection of siderophore production by fungal isolates, CAS agar plates were prepared by dissolving 100 mL of CAS-HDTMA reagent into 900 mL of molten potato dextrose agar. A fungal disc of 5 mm diameter was cut from a 7-day-old culture and aseptically placed at the centre of each petri plate. Plates without fungal disc serve as a control. All plates were incubated for 5–7 days at 28 °C, and the formation of an orange zone around the fungal mycelium indicated positive siderophore production^36^.

### Antioxidant activity of fungal crude metabolites

#### 2,2-diphenyl-1-picrylhydrazyl (DPPH) scavenging assay

The DPPH radical scavenging activity of the ethyl acetate extract of isolated endophytic fungi was adopted from Almustafa and Yehia^37^ with minor modifications. To prepare a 0.1 mM DPPH solution, 3.94 mg of DPPH was dissolved in 100 mL of methanol and kept in the dark for 1 h; the absorbance was then adjusted to 0.9–1.0 at 517nm. Then, 1 mL of different concentrations (5, 25, 50, 100, 200 µg mL^−1^) of fungal extract was added to 1 mL of DPPH solution, and the mixture was incubated in the dark for 30 minutes. Control was maintained with DPPH and methanol. After incubation, absorbance was taken at 517 nm. Ascorbic acid was used as a positive control. The experiment was conducted in triplicate. The DPPH scavenging potential of fungal extract was calculated using the following formula:

$$\mathrm{S}\mathrm{c}\mathrm{a}\mathrm{v}\mathrm{e}\mathrm{n}\mathrm{g}\mathrm{i}\mathrm{n}\mathrm{g} \mathrm{\%} = [(\mathrm{A}0 - \mathrm{A}\mathrm{s}) / \mathrm{A}0] \times 100$$

Where, A_0_ = Absorbance of control.

As = Absorbance of sample.

The concentrations of the tested sample that scavenge ≥50% of DPPH radicals (IC_50_) were determined by linear interpolation.

#### 2,2-azino-bis-3-ethylbenzothiazoline-6-sulfonic acid (ABTS*)* scavenging assay

The ABTS radical-scavenging activity of fungal metabolites was assessed using the method proposed by Hameed et al.^38^, with minor modifications. Briefly, different concentrations (5–200 µg mL^−1^) of fungal crude mixed with 7.00 mM stock solution of ABTS^+^, and incubated at dark for 30 min. Ascorbic acid solution was used as a standard positive control, and the sample absorbance was measured at 734 nm. The percentage scavenging of ABTS^+^ was determined as:

$$\mathrm{S}\mathrm{c}\mathrm{a}\mathrm{v}\mathrm{e}\mathrm{n}\mathrm{g}\mathrm{i}\mathrm{n}\mathrm{g} \mathrm{\%} = [(\mathrm{A}_0 - \mathrm{A}\mathrm{s}) / \mathrm{A}_0] \times 100$$

where, A_0_ = absorbance of control

As = absorbance of sample.

The concentrations of the tested sample that scavenge ≥50% of ABTS^+^ radicals (IC_50_) were determined by linear interpolation.

### *In vivo* assessment of fungal endophytes on maize plant growth promotion

#### Preparation of fungal inoculum

To prepare the fungal inoculum of three isolated endophytic fungi, CJR-1, CJR-2, and CJR-4, firstly inoculated onto potato dextrose agar (PDA) media and incubated at 28 °C for seven days in a BOD incubator. Mycelial plugs of each fungus were aseptically transferred to sterilized sorghum seeds and incubated until full colonization was observed^39^. After 14 days, sorghum seeds colonized with endophytic fungi were used as an inoculum for plant growth assessment.

#### Pot experiment

A pot experiment was conducted to evaluate the ability of isolated fungal endophytes to promote maize plant growth. The maize seeds were disinfected by soaking in 2.5% sodium hypochlorite for 5 minutes, followed by immersion for 1 min. in 70% ethanol and washed 5 times with sterile distilled water. To assess the effectiveness of surface sterilization, 100 µL of the final rinse water was plated onto PDA medium and incubated. Then 5 seeds were sown in each pot (16 × 17 cm) filled with three times autoclaved 90% gardening soil and 10% coco peat. After 10 days, three healthy, equal sized plants were selected, and the rest were removed from the pot. Further, two sorghum seeds colonized with endophytic fungi were placed near each maize root by making a hole. Control was introduced with non-infected sorghum seeds, whereas the positive control was treated with commercially available AMF granules (Apna Power Mycorrhizal Biofertilizer, Hindustan Urvarak & Rasayan Limited, India). Each treatment was conducted in triplicate, with three plants per pot.

Plants were irrigated with tap water and kept under natural sunlight conditions for 28 days after treatment (DAT). At the end of the experiment, the plants were carefully harvested and washed thoroughly under running tap water to remove adhering soil particles. Plant growth parameters, including root and shoot length, were recorded. Fresh biomass of roots and shoots was determined immediately after harvest. For dry biomass determination, the harvested plant samples were oven-dried at 50–60°C for 24 - 48 h until a constant weight was reached, and the dry weight was recorded using an analytical balance (Biogen Scientific). Photosynthetic pigments, including chlorophyll a, Chlorophyll b, total chlorophyll, and carotenoids, were also quantified.

For the estimation of photosynthetic pigments, 0.5 gm of fresh leaves was ground with 80% aqueous acetone using a mortar and pestle, then filtered through Whatman No. 1 filter paper. Then, using 80% acetone, the filtrate was made up to 100 mL, and the absorbance was measured at 665, 649, and 470 nm in a spectrophotometer^40^. The concentrations of chlorophyll a, chlorophyll b, and carotenoid in maize leaves were determined using the following equations: mg chlorophyll (a)/gm tissue = 11.63(A665) – 2.39(A649), mg chlorophyll (b)/gm tissue = 20.11(A649) – 5.18(A665) and carotenoids mg/gm fresh weight = 100 X O.D _470_ – 1.82 C_a_ – 85.02 C_b_/198. Where “A” denotes the absorbance.

### Assessment of root colonization potential of *Aspergillus cf. terreus* CJR-4

Based on preliminary morphological observations of maize plants following fungal inoculation, *A. cf. terreus* CJR-4 inoculated plants were selected for assessment of root colonization. Maize roots were sampled at 14 and 28 days after treatment (DAT).

Root tissues were thoroughly washed under running tap water for 3–5 times to remove adhering soil particles. The root segments were surface-sterilized by immersion in 70% ethanol for 2 min, followed by 2% sodium hypochlorite for 3 min, and rinsed 3–4 times with sterile distilled water. Sterilized roots were dried on sterile tissue paper under a laminar airflow and cut into 1 cm segments. Twenty root segments were aseptically placed on PDA medium supplemented with streptomycin sulphate (30 mg/L) to inhibit the bacterial growth and incubated at 28 °C for 7 days in a BOD incubator. To ensure surface sterilization, 100 µL of the final rinse water was pipetted onto PDA and incubated under the same conditions. Emerging fungal colonies from root segments were identified based on their colony morphology and microscopic features and compared with the original inoculated fungal isolate. Colonization frequency was calculated using the following formula^41^ as:

$$\mathrm{C}\mathrm{o}\mathrm{l}\mathrm{o}\mathrm{n}\mathrm{i}\mathrm{z}\mathrm{a}\mathrm{t}\mathrm{i}\mathrm{o}\mathrm{n}\:\: \mathrm{f}\mathrm{r}\mathrm{e}\mathrm{q}\mathrm{u}\mathrm{e}\mathrm{n}\mathrm{c}\mathrm{y}\:\: (\mathrm{\%}) = (\mathrm{N}\mathrm{u}\mathrm{m}\mathrm{b}\mathrm{e}\mathrm{r}\:\: \mathrm{o}\mathrm{f}\:\: \mathrm{r}\mathrm{o}\mathrm{o}\mathrm{t}\:\: \mathrm{s}\mathrm{e}\mathrm{g}\mathrm{m}\mathrm{e}\mathrm{n}\mathrm{t}\mathrm{s}\:\: \mathrm{c}\mathrm{o}\mathrm{l}\mathrm{o}\mathrm{n}\mathrm{i}\mathrm{z}\mathrm{e}\mathrm{d} / \mathrm{T}\mathrm{o}\mathrm{t}\mathrm{a}\mathrm{l}\:\: \mathrm{r}\mathrm{o}\mathrm{o}\mathrm{t}\:\: \mathrm{s}\mathrm{e}\mathrm{g}\mathrm{m}\mathrm{e}\mathrm{n}\mathrm{t}\mathrm{s}\:\: \mathrm{e}\mathrm{x}\mathrm{a}\mathrm{m}\mathrm{i}\mathrm{n}\mathrm{e}\mathrm{d}) \times 100$$

In addition, further root colonization was confirmed by microscopic examination of stained root tissues following the method of Lekshmi et al.^42^ with minor modifications. Briefly, roots were collected at 28 DAI, thoroughly washed with running tap water, cut into approximately 1 cm in segments. The root segments were immersed in 10% KOH at 80–90 °C for 10 min, rinsed 2–3 times with distilled water, and acidified in 1 M HCL for 5 min. Decanted HCl and immediately stained with lactophenol cotton blue for 15 min, followed by washing with distilled water to remove excessive stain. The stained root segments were mounted on slides and examined under a compound microscope (10 × and 40 × magnification).

### Statistical data analysis

Experimental data were analyzed using one-way ANOVA followed by Tukey’s multiple comparisons test using GraphPad Prism software (version 10.4.2). Results were expressed as mean ± standard deviation (n=3). Results with a p*-*value less than 0.05 (p < 0.05) were considered statistically significant.

## Results

### Isolation and identification of fungal endophytes

**Fig. 1:**
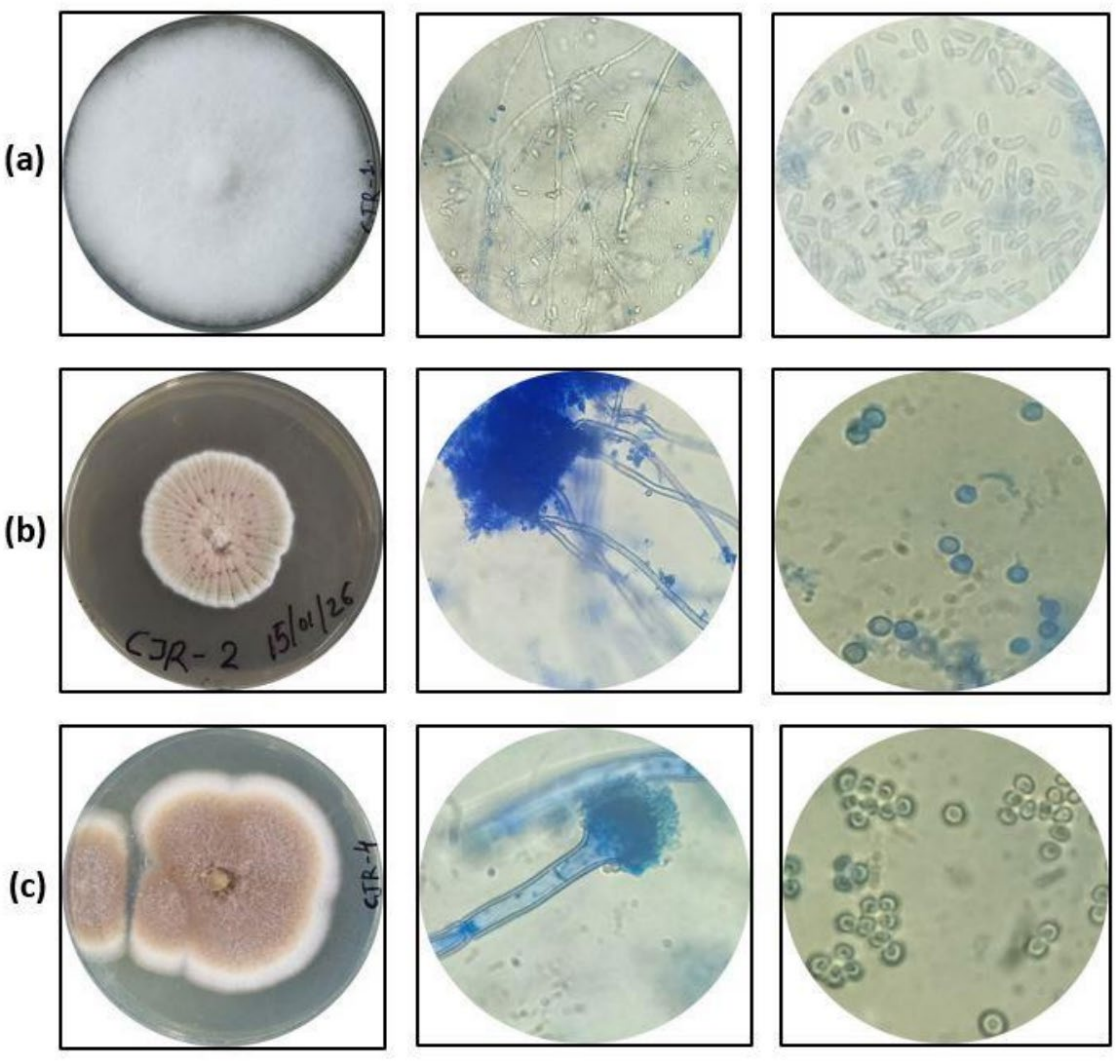
Isolated endophytic fungi grown on PDA medium. (**a**) CJR-1: colony morphology, hyphae, and conidial structure. (**b**) CJR-2: colony morphology, conidiophores, and globose conidia. (**c**) CJR-4: colony morphology, conidiophore in vesicle with phialides and conidia.

**Fig. 2:**
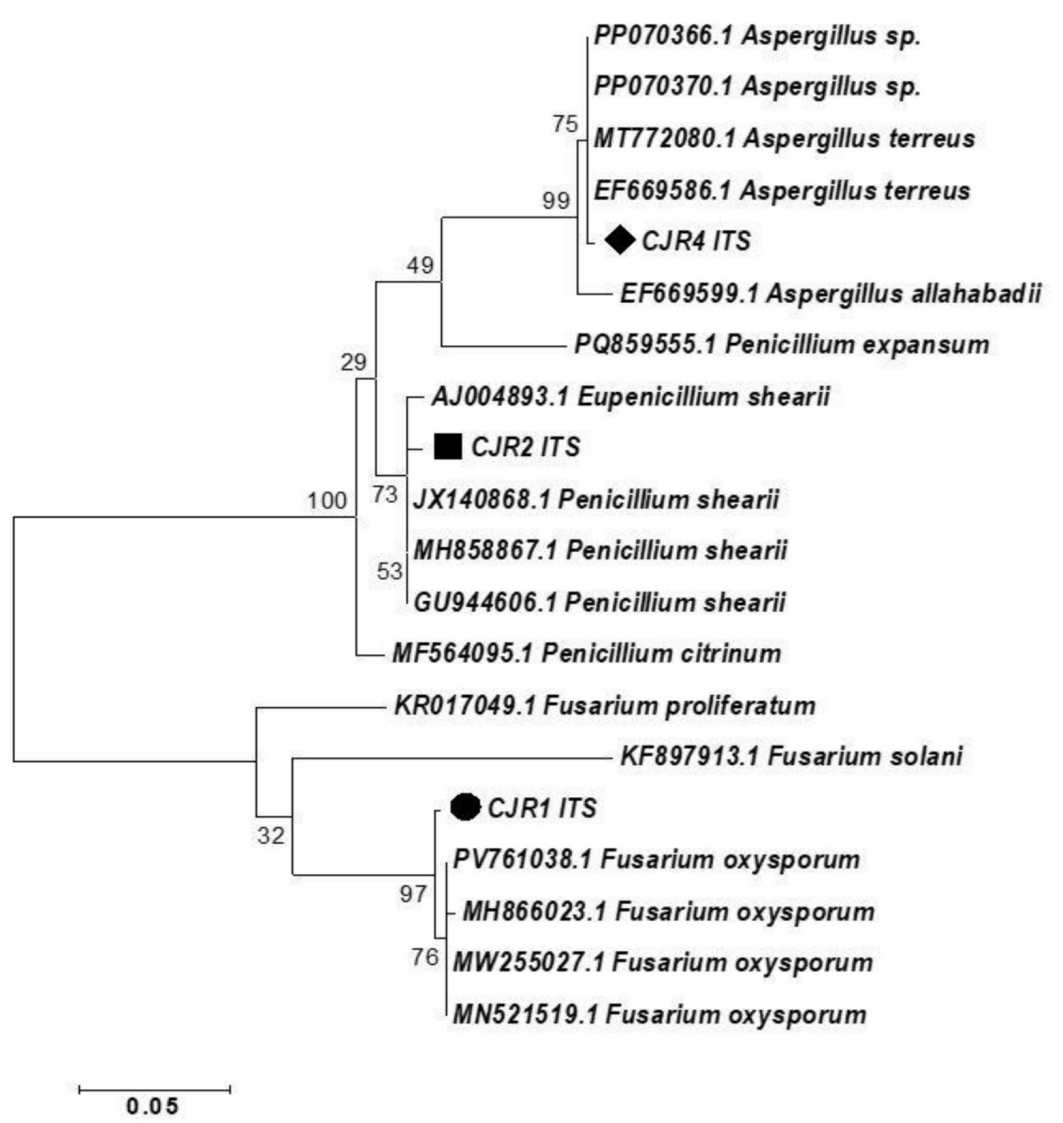
Maximum Likelihood phylogenetic tree based on ITS rDNA sequences showing the relationships of the fungal isolates CJR-1, CJR-2, and CJR-4 with closely related reference taxa retrieved from NCBI GenBank. The tree was constructed with MEGA 6 using Tamura-Nei model with bootstrap replicates of 1000.

Three morphologically distinct fungal endophytes were isolated from the inner root tissues of *Crotalaria juncea* L. (Fig. 1). The isolates were initially characterized based on macroscopic and microscopic features, including colony morphology, pigmentation, conidiophore structure, and conidial characteristics. Isolate CJR-1 initially produced white colonies that developed pink pigmentation during later growth stages. Microscopic examination revealed septate hyphae with micro- and macroconidia, along with chlamydospores, as characteristic features of *Fusarium oxysporum*. Isolate CJR-2 produced velvety to powdery green colonies with green sporulation at colony margins. Microscopic structures showed branched conidiophores with metulae and phialides bearing globose conidia, morphologically similar to those of *Penicillium* spp. In contrast, CJR-4 developed orange-brown to light-brown powdery colonies over time. Microscopic examination revealed conidiophores terminating in globose vesicles. Conidia are globular to ellipsoidal, smooth-walled, and uninucleate. These morphological characteristics were consistent with those reported for *Aspergillus terreus*. Furthermore, molecular identification was performed using ITS rDNA sequence analysis. Total genomic DNA of the isolated endophytic fungi was extracted and analyzed for their ITS region. The PCR products from fungal isolates were sequenced and analyzed using BLAST against the NCBI GenBank database. BLAST analysis revealed that CJR-1 exhibited 99.32% sequence identity with *Fusarium oxysporum* (Accession No. PV761038.1) and clustered within the *F. oxysporum* lineage with strong bootstrap support (97%), thereby supporting its identification as *Fusarium oxysporum* (Accession No. PZ477045). CJR-2 showed 95.19% sequence identity with *Penicillium shearii* (GenBank Accession No. JX140868.1) and formed a moderately supported clade (73%), supporting its identification as *Penicillium* sp. (GenBank Accession No. PZ477429). Similarly, CJR-4 exhibited 97.15% sequence identity with *Aspergillus terreus* (Accession No. MT772080.1) and clustered within the *A. terreus* clade with strong bootstrap support (99%), supporting its designation as *Aspergillus cf. terreus* (GenBank Accession No. PZ477430). The ITS sequences generated in the present study were deposited in GenBank under accession numbers PZ477045 (CJR-1), PZ477429 (CJR-2), and PZ477430 (CJR-4). Multiple sequence alignment was performed using ClustalW, and a maximum-likelihood phylogenetic tree was constructed in MEGA 6 software using the Tamura-Nei model with 1,000 bootstrap replicates. Overall, the phylogenetic tree analysis confirmed the placement of the isolated endophytic fungi within the genera *Fusarium*, *Penicillium*, and *Aspergillus* (Fig. 2).

### *In vitro* antagonistic activity of fungal isolates against phytopathogenic fungi

**Fig. 3:**
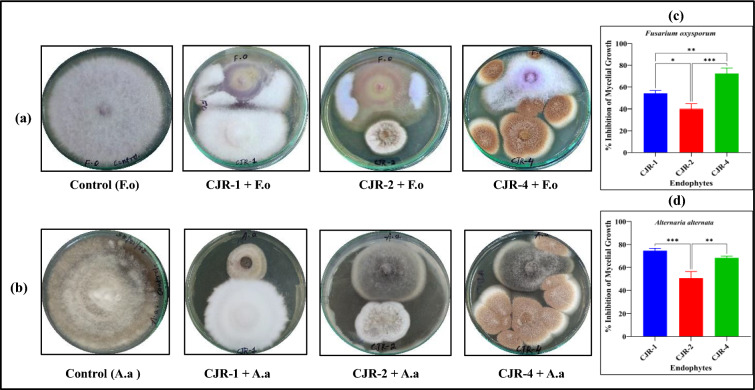
Antagonistic activity of endophytic fungi against (**a**) *F. oxysporum*, (**b**) *A. alternata*. Bar graph of (**c**) and (**d**) showing the comparative % mycelial inhibition of endophytic fungi against tetsed pathogens. Asterisks indicate significant statistical variation at p < 0.05. Data are represented as mean ± SD (n=3).

The antagonistic activities of fungal isolates CJR-1, CJR-2, and CJR-4 were evaluated against phytopathogenic fungi *F. oxysporum* and *A. alternata* using the dual-culture method, as shown in Fig. 3. The antagonistic potential of fungal isolates CJR-1 and CJR-4 was found to be effective against both tested fungal pathogens, as depicted in Table 1. Among the fungal isolates, *A. cf. terreus* CJR-4 exhibited strong antagonistic activity against both the fungal pathogens, resulting in mycelial growth inhibition of 72.41 ± 5.02% against *F. oxysporum* and 68.31 ± 1.53% against *A. alternata*. In contrast, *F. oxysporum* CJR-1 exhibited the highest mycelial inhibition (74.60 ± 2.06%) against *A. alternata*.

**Table 1:** Effect of endophytic fungal isolates on mycelial growth of tested pathogens. Data are represented by three replicates with ± SD (n=3).

**Fungal endophytes**	**Percentage inhibition of pathogens mycelial growth**	
	*Fusarium oxysporum*	*Alternaria alternata*
*Fusarium oxysporum* CJR-1	54.15 ± 2.66	74.60 ± 2.06
*Penicillium* sp*.* CJR-2	40.10 ± 4.44	50.66 ± 5.70
*Aspergillus cf. terreus* (CJR-4)	72.41 ± 5.02	68.31 ± 1.53

### Antifungal activity of ethyl acetate crude metabolites

**Fig. 4:**
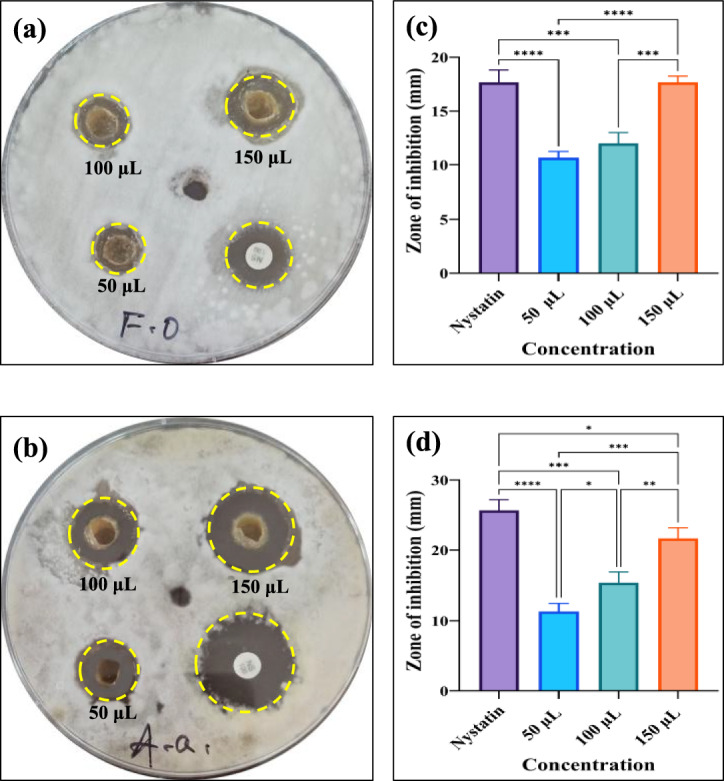
Antifungal activity of fungal crude metabolites produced by *A. cf. terreus* CJR-4 against phytopathogenic fungi. (**a**, **b**) Images showing the zones of inhibition produced by fungal crude metabolites against *F. oxysporum* and *A. alternata*. (**c**-**d**) Bar graph showing comparative inhibition zones of tested fungal pathogens at different concentrations of crude metabolites and the positive control (Nystatin). Data represent as mean ± SD of n=3. Asterisks indicate the statistical variation at p < 0.05.

The ethyl acetate crude extract of *A. cf. terreus* CJR-4 exhibited antifungal activity against both tested phytopathogenic fungi, *F. oxysporum* and *A. alternata*, as evidenced by the formation of distinct zones of inhibition (ZOI) (Fig. 4a, b). The crude metabolites exhibited a dose-dependent antifungal effect. Among the tested concentrations, the highest zone of inhibition (21.66 ± 1.52 mm) was observed against *A. alternata,* followed by *F. oxysporum* (15.33 ± 1.15 mm) at 150 µL of crude extract. On the other hand, positive control, Nystatin produced inhibition zones of 25.66 ± 1.52 mm and 17.66 ± 1.54 mm against *A. alternata* and *F. oxysporum* (Fig. 4c, d; Table 2).

**Table 2:** Antifungal activity of fungal crude metabolites against fungal pathogens. Data are represented by three replicates with ± SD (n=3).

Phytopathogenic fungi	Antifungal activity of crude metabolites (ZOI in mm)			
	NS 100	50 µL	100 µL	150 µL
*Fusarium oxysporum*	17.66 ± 1.54	10.66 ± 0.57	12.00 ± 1.00	15.33 ± 1.15
*Alternaria alternata*	25.66 ± 1.52	11.33 ± 1.15	15.33 ± 1.52	21.66 ± 1.52

### GC-MS screening for secondary metabolites of ethyl acetate extract of fungal isolate *Aspergillus cf. terreus* CJR-4

**Fig. 5:**
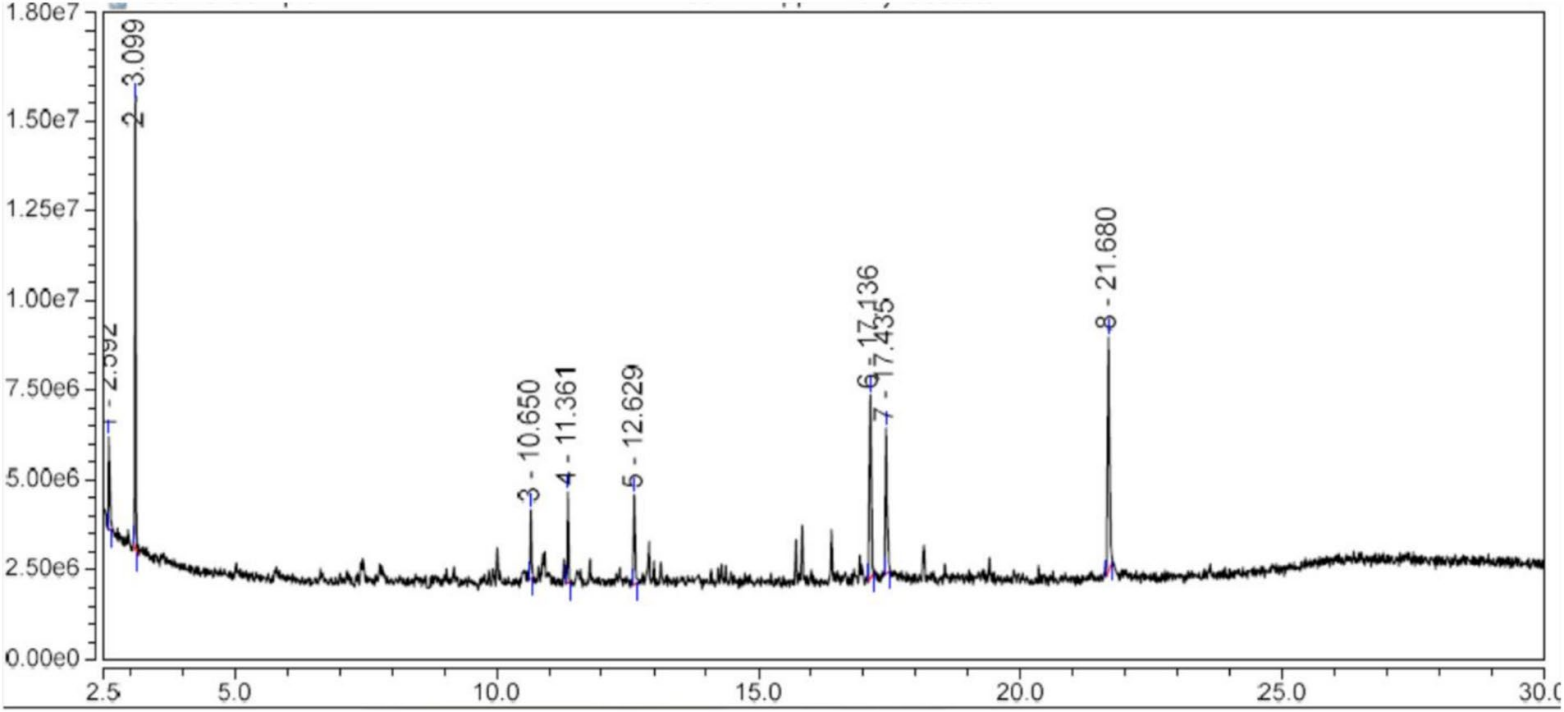
GC-MS chromatograms of ethyl acetate extract of CJR-4 isolates.

Endophytic fungi secrete various secondary metabolites to facilitate growth and defense against pathogens. The identification of those bioactive secondary metabolites is crucial for understanding their functions and discovering compounds with promising benefits. As *A. cf. terreus* CJR-4 showed potent antagonistic activity against both phytopathogens, an attempt was made to analyze its chemical composition using GC-MS, which revealed several compounds with potent biological roles (Fig. 5; Table 3). Among the detected compounds, the major ones by peak area percentage were 1,3,5-cycloheptatriene (29.29%), diphenyl sulphone (22.15%). Other detected compounds such as o-tert-Butylhydroxylamine (13.48%), propane, 2-methyl-1-nitro- (11.77%), 1H-Indene, 1-methylene- (6.38%), 2-Nonen-1-ol (6.04%), ethane, 1,1-diethoxy- (5.77%) and Ethanone,2-(formyloxy)−1-phenyl- (5.10%).

**Table 3:** Chemical profile of ethyl acetate extract of CJR-4 isolate (major chemical constituents).

**Sl. no**	**Retention time (RT)**	**Compounds name**	**Relative area%**	**Molecular weight**	**Formula**
01	2.592	Ethane, 1,1-diethoxy-	5.77	118.17	C_6_H_14_O_2_
02	3.099	1,3,5-Cycloheptatriene	29.29	92.14	C_7_H_8_
03	10.650	Ethanone,2-(formyloxy)−1-phenyl-	5.10	164.16	C_9_H_8_O_3_
04	11.361	2-Nonen-1-ol	6.04	142.24	C_9_H_18_O
05	12.629	1H-Indene, 1-methylene-	6.38	128.17	C_10_H_8_
06	17.136	o-tert-Butylhydroxylamine	13.48	89.14	C_4_H_11_NO
07	17.435	Propane, 2-methyl-1-nitro-	11.77	103.12	C_4_H_9_NO_2_
08	21.680	Diphenyl sulfone	22.15	218.27	C_12_H_10_O_2_S

### Indole-3-acetic acid production (IAA)

**Fig. 6:**
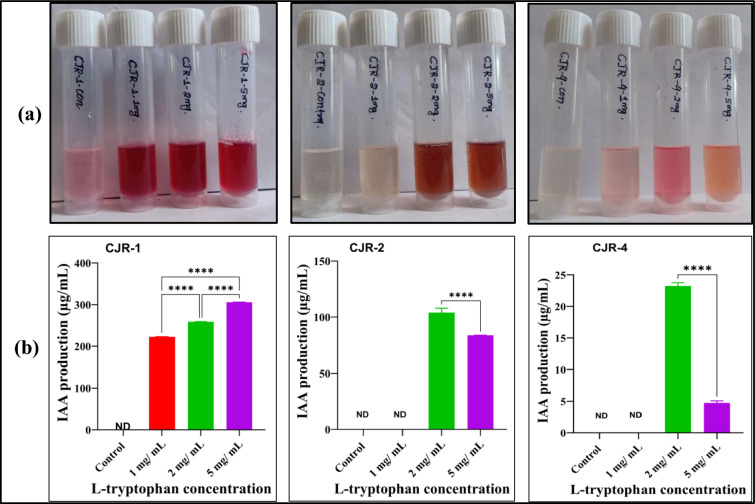
(**a**) Qualitative determination of IAA production by fungal endophytes; (**b**) quantitative estimation of IAA production by fungal endophytes. Data are represented as mean ± SD of n=3. Asterisks **** represented the significant statistical variation at the p < 0.0001 level.

Phytohormones play a crucial role in plant growth and development. In this study, IAA production by fungal endophytes was determined under different concentrations of L-tryptophan as a precursor. Among the three isolates, CJR-1 produced the highest IAA concentration (305.69 ± 0.41 µg/mL) at 5 mg/mL L-tryptophan (Fig. 6a, b). In contrast, isolates CJR-2 and CJR-4 exhibited maximum IAA production at 2 mg/mL of L-tryptophan, yielding 104.05 ± 3.89 and 23.22 ± 0.52 µg/mL, respectively. Interestingly, IAA production by isolates CJR-2 and CJR-4 decreased at 5 mg/mL of L-tryptophan, suggesting that IAA biosynthesis in these endophytes was influenced by the precursor concentration (Table 4).

**Table 4:** Quantitative estimation of IAA production by isolated fungal endophytes.

**Tryptophan conc. (mg/mL)**	**IAA production by fungal endophytes (µg/mL)**		
	CJR-1	CJR-2	CJR-4
1 mg/mL	222.49 ± 0.34	0 ± 0	0 ± 0
2 mg/mL	258.68 ± 0.39	104.05 ± 3.89	23.22 ± 0.52
5 mg/mL	305.69 ± 0.41	83.70 ± 0.07	4.74 ± 0.32

### Phosphate solubilization

**Fig. 7:**
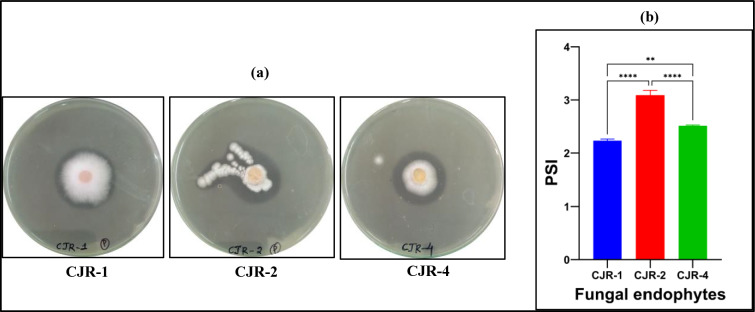
Fungal endophytes growing on PVK agar medium. (**a**) Clear halo zone around the fungal colony represents phosphate solubilization ability; (**b**) comparative PSI of fungal isolates. Data represent the mean ± SD of n=3. Asterisks ** & **** represent statistical variation at p < 0.0021 & 0.001 level.

To determine phosphate solubilization potential, fungal endophytes were inoculated on PVK agar medium, and all isolates exhibited positive phosphate solubilizing activities indicated by the formation of clear zones around their growth on medium (Fig. 7). Among three isolates, the highest phosphate solubilization index occurred with CJR-2 as 3.09 ± 0.09, followed by CJR-4 (2.51 ± 0.01) and CJR-1 (2.23 ± 0.03) (Table 5; Fig. 7b).

**Table 5:** Phosphate solubilization index of fungal isolates.

**Fungal endophytes**	**Phosphate solubilization index**
*Fusarium oxysporum* CJR-1	2.23 ± 0.03
*Penicillium* sp*.* CJR-2	3.09 ± 0.09
*Aspergillus cf. terreus* CJR-4	2.51 ± 0.01

### Ammonia production

**Fig. 8:**
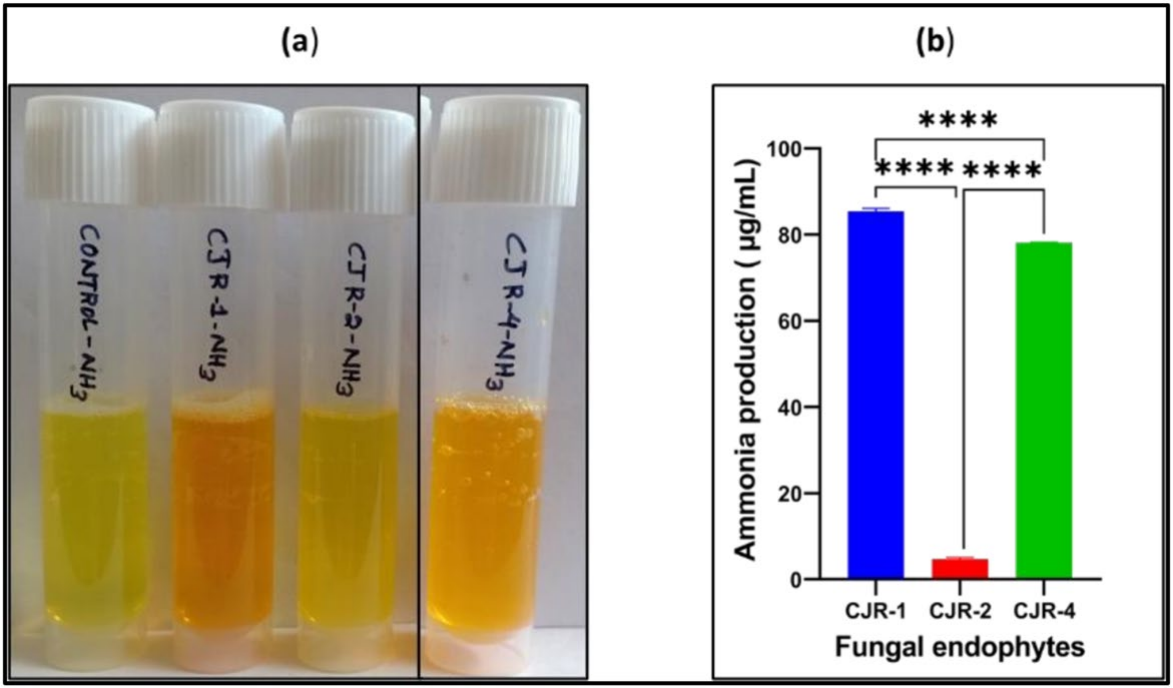
Determination of ammonia production by Nessler’s test. (**a**) Qualitative determination of ammonia production; (**b**) quantitative estimation of ammonia production. Asterisks **** reflect the significant statistical variation at the p < 0.0001 level.

The ammonia produced by endophytic fungi plays a crucial role in soil fertility and nitrogen recycling. Further, they regulate soil pH levels by secreting acidic substances during ammonia production. All three tested endophytes exhibited colour changes from pale yellow to brown, indicating that they can produce ammonia. The intensity of colour changes indicates the degree of ammonia production, as shown in Fig. 8a. The spectrophotometric analysis of the tested solution exhibited that CJR-1 produced a significantly high concentration of ammonia, i.e., 85.44 ± 0.63 µg/mL (Fig. 8b), followed by CJR-4 as 78.07 ± 0.17 µg/mL. In contrast, lower ammonia production was observed in CJR-2 isolates (Table 6).

**Table 6:** Quantitative estimation of ammonia production by isolated fungal endophytes.

**Fungal endophytes**	**Ammonia production (µg/mL)**
*Fusarium oxysporum* CJR-1	85.44 ± 0.63
*Penicillium* sp*.* CJR-2	4.73 ± 0.34
*Aspergillus cf. terreus* CJR-4	78.07 ± 0.17

### Siderophore production

**Fig. 9:**
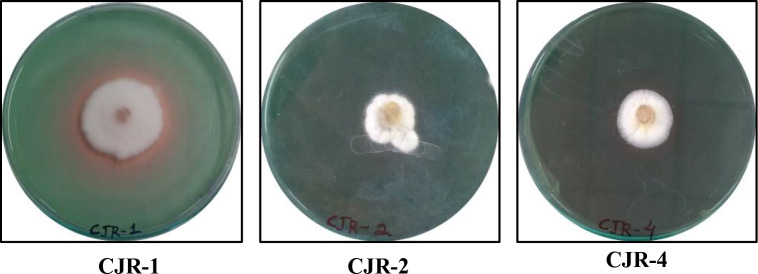
Qualitative determination of the siderophore test in CAS agar medium.

The ability of the tested endophytes to change colour from blue to orange in CAS agar indicates siderophore production. The result showed that CJR-1 exhibited the formation of an orange halo zone around the fungal colony, whereas CJR-2 and CJR-4 resulted in a negative siderophore test (Fig. 9; Table 7).

**Table 7:** Qualitative determination of siderophore production by fungal endophytes.

**Fungal endophytes**	**Quantitative siderophore test**
*Fusarium oxysporum* CJR-1	+
*Penicillium* sp*.* CJR-2	-
*Aspergillus cf. terreus* CJR-4	-

### DPPH and ABTS radical scavenging activity

**Fig. 10:**
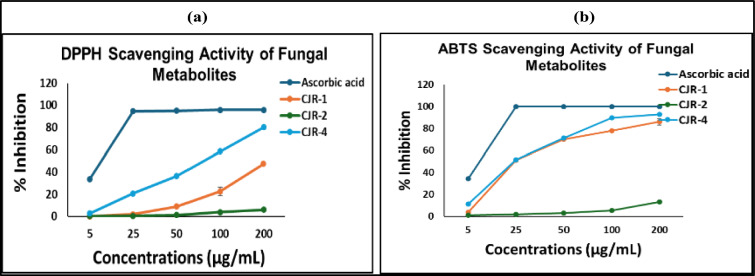
**(a**, **b**) Representing DPPH and ABTS radical scavenging activity of different concentrations of ascorbic acid and fungal crude metabolites.

The ethyl acetate extract of three fungal isolates exhibited dose-dependent free radical scavenging activity (Fig. 10a, b). Fungal extract CJR-4 showed outstanding percentage scavenging potential of DPPH, ranging from 2.78 ± 0.08 to 80.09 ± 0.11, and ABTS 11.35±0.12 to 92.66±0.17 at the concentration of 5–200 µg/mL (Table 8). On the other hand, CJR-2 showed lower scavenging activity than CJR-1. Furthermore, the CJR-4 extract showed IC_50_ values of 80.85 µg/mL (DPPH) and 24.27 µg/mL (ABTS^+^), respectively (Table 9).

**Table 8:** DPPH and ABTS^+^ scavenging activity of ethyl acetate extract of fungal metabolites.

**Concentration**	**Inhibition percentage**							
	Ascorbic acid		CJR-1		CJR-2		CJR-4	
	DPPH	ABTS	DPPH	ABTS	DPPH	ABTS	DPPH	ABTS
5 µg/mL	33.53±0.00	34.34± 0.14	0.13±0.12	3.61±0.27	0.33±0.11	0.92±0.15	2.78±0.08	11.35±0.12
25 µg/mL	94.51±0.05	100.00± 0.0	1.95±0.11	51.30±0.18	0.60±0.20	1.94±0.46	20.82±0.11	51.45±0.32
50 µg/mL	95.12±0.05	100.00± 0.0	8.93±0.14	69.88±0.62	1.34±0.12	3.05±0.26	36.35±0.03	71.05±0.47
100 µg/mL	95.93±0.04	100.00± 0.0	22.54±3.94	77.91±0.50	3.76±0.12	5.18±0.29	58.46±0.03	89.64±0.14
200 µg/mL	95.93±0.04	100.00± 0.0	47.38 ± 0.36	85.94±0.67	6.05±0.22	12.97±3.01	80.09±0.11	92.66±0.17

**Table 9:** IC_50_ values of ascorbic acid and fungal crude metabolites in DPPH and ABTS^+^ assay.

**Samples**	**DPPH IC**_**50**_** (µg/mL)**	**ABTS IC**_**50**_** (µg/mL)**
Ascorbic acid	10.40	9.77
*Fusarium oxysporum* CJR-1	>200	24.45
*Penicillium* sp*.* CJR-2	>200	>200
*Aspergillus cf. terreus* CJR-4	80.85	24.27

### The assessment of growth promotion in maize plants by inoculation of endophytic fungal isolates and AMF in pot conditions

**Fig. 11:**
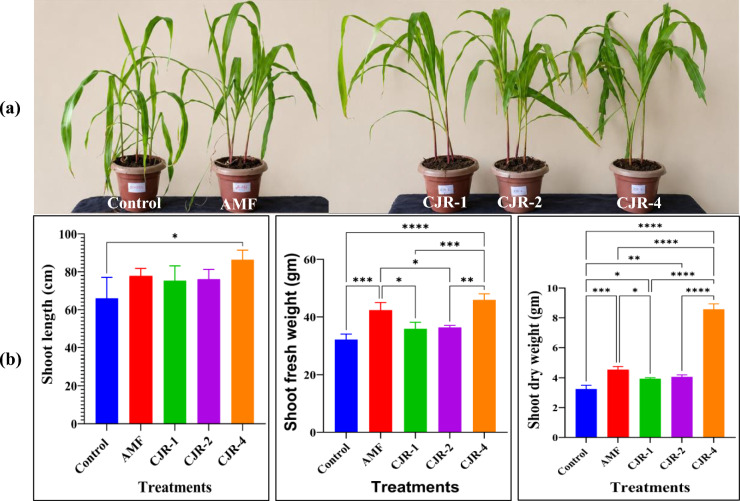
Plant growth promotion test. (**a**) Effect of endophytic fungal inoculum and AMF on growth promotion of maize plant, (**b**) comparative bar graph of shoot length and shoot biomass among the treatments. Data represent triplicate values of mean ± SD. Asterisks ** & **** represent statistical variation at p < 0.0021 & 0.001 level.

**Fig. 12:**
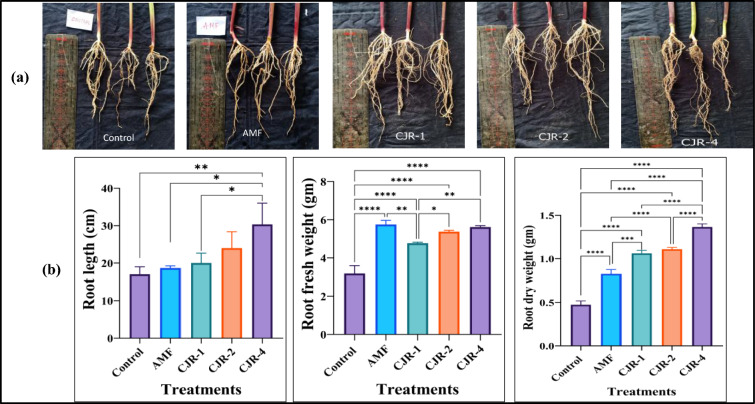
(**a**) Showing the effect of root length among the treatments and control, (**b**) effect of inoculation on root length, fresh weight, and dry weight. Asterisks indicate significant statistical variation at the p < 0.05 level.

A pot experiment was conducted to investigate the effects of isolated endophytic fungi (CJR-1, CJR-2, and CJR-4) and AMF on the growth promotion of maize plants (Fig.11,12). Maize plants treated with endophytic fungi and AMF showed significant improvements (p < 0.05) in overall growth and biomass compared with the control (Table 10). The control treatment resulted in the lowest root and shoot lengths of 17.0 ± 2.0 cm and 66.04 ± 11.07 cm, followed by the fresh and dry mass values. Plant treated with AMF exhibited the highest root fresh weight (5.75 ± 0.21 gm) and enhanced overall growth. The endophytic isolates CJR-1 and CJR-2 also showed a significant increase in overall growth parameters compared with the control. On the other hand, the most pronounced growth was observed under treatment with CJR-4 isolates, which exhibited the highest root length (30.33 ± 5.68 cm) and shoot length (86.36 ± 5.08 cm). Similarly, it significantly (p < 0.05) increased fresh shoot biomass (45.93 ± 2.08 gm) and dry mass of both shoots (8.57 ± 0.35 gm) and roots (1.36 ± 0.03 gm), representing substantial growth performance compared to AMF, CJR-1, CJR-2, and the control treatments.

**Table 10:** Assessment of growth promotion in maize plants inoculated with endophytic fungi and AMF. Values are represented as mean ± SD of a triplicate experiment.

**Treatment**	**Plant height (cm)**		**Fresh biomass (gm)**		**Dry biomass (gm)**	
	**Root length**	**Shoot length**	**Root weight**	**Shoot weight**	**Root weight**	**Shoot weight**
Control	17.0 ± 2.0	66.04 ± 11.07	3.17 ± 0.41	32.10 ± 1.90	0.47 ± 0.04	3.25 ± 0.25
AMF	18.66±0.57	77.89 ± 3.87	5.75 ± 0.21	42.38 ± 2.61	0.82 ± 0.04	4.66 ± 0.10
CJR-1	20.0 ± 2.64	75.35 ± 7.75	4.76 ± 0.04	35.85 ± 2.27	1.06 ± 0.03	3.94 ± 0.06
CJR-2	24.0 ± 4.35	76.2 ± 5.08	5.37 ± 0.07	36.34 ± 0.68	1.10 ± 0.02	4.06 ± 0.13
CJR-4	30.33± 5.68	86.36 ± 5.08	5.61 ± 0.07	45.93 ± 2.08	1.36 ± 0.03	8.57± 0.35

### Effects of endophytic fungal inoculum and AMF on chlorophyll and carotene content

**Fig. 13:**
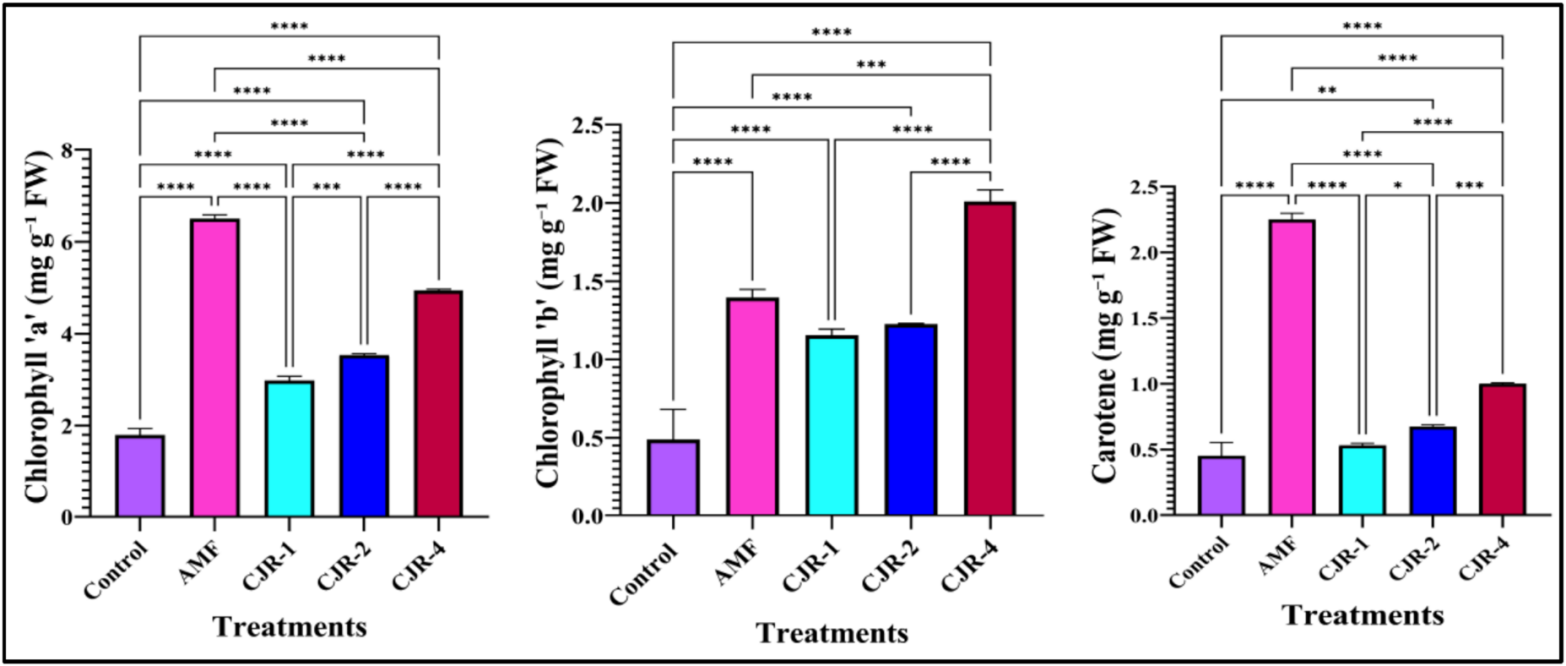
Accumulation of photosynthetic pigments chlorophyll a, chlorophyll b, and carotene in control and treatment groups. Asterisks indicate significant statistical variation at the p < 0.05 level.

Photosynthetic pigment levels across the different treatments showed significant variation (Fig. 13). The plant inoculated with AMF resulted in the highest accumulation of photosynthetic pigment, chlorophyll a (6.50 ±0.07 mg g⁻^1^ FW), chlorophyll b (1.39 ± 0.05 mg g⁻^1^ FW), and carotene (2.24± 0.04 mg g⁻^1^ FW) along with total chlorophyll (a+b) 7.89 ± 0.13 mg g⁻^1^ FW, (Table 11).

**Table 11:** Depicted the effect of fungal endophytes and AMF on chlorophyll and carotene accumulation in maize plants.

**Treatments**	**Chlorophyll ‘a’ mg g**^**-1**^** FW**	**Chlorophyll ‘b’ mg g**^**-1**^** FW**	**Carotene** **mg g**^**-1**^** FW**	**Chlorophyll ‘a+b’ mg g**^**-1**^** FW**
Control	1.79 ±0.14	0.48 ±0.19	0.45 ±0.10	2.28 ±0.33
AMF	6.50 ±0.07	1.39 ±0.05	2.24 ±0.04	7.89 ±0.13
CJR-1	2.97 ±0.09	1.15 ±0.03	0.53 ±0.01	4.13 ±0.13
CJR-2	3.53 ±0.03	1.22 ± 0.00	0.67 ±0.01	4.75 ±0.03
CJR-4	4.93 ±0.03	2.00 ±0.07	1.00 ±0.00	6.94 ±0.10

Among the treatments of isolated endophytes, CJR-4 exhibited maximum accumulation of measurable pigment with chlorophyll a (4.93 ±0.03 mg g⁻^1^ FW), followed by chlorophyll b (2.00± 0.07 mg g⁻^1^ FW), total chlorophyll (a+b) as 6.94 ± 0.10, and carotene (1.00 ± 0.00 mg g⁻^1^ FW). At the same time, CJR-2 showed higher accumulation of all these pigments than CJR-1.

All the plants inoculated with fungal isolates showed a significant (p < 0.05) increase in photosynthetic pigments compared to the control.

### Root colonization potential of fungal isolate *Aspergillus cf. terreus* CJR-4

**Fig. 14:**
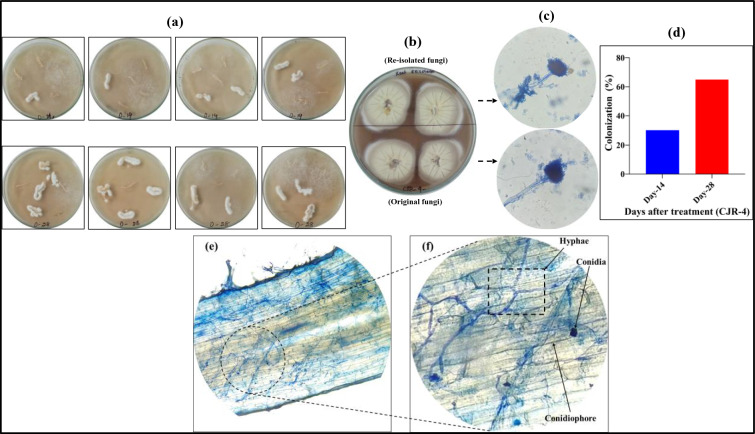
Colonization of *A. cf. terreus* CJR-4 in maize roots. (**a**) Re-isolation of CJR-4 fungus from surface-sterilized root segments at 14 and 28 days after treatment (DAT). (**b**-**c**) Morphological and microscopic comparison of treated CJR-4 isolate with re-isolated fungus from maize roots, showing similar colony morphology, pigmentation, conidiophores, conidial heads, and conidia. (**d**) Bar graph showing the colony frequency (%) of treated CJR-4 isolate in maize roots at 14 and 28 DAT. (**e**-**f**) Microscopic examination of maize root tissues (10 × and 40 × magnification) shows fungal hyphae, conidiophores, and conidia, confirming successful colonization of the CJR-4 isolate. Data represented as mean ± SD (n=3).

Root colonization of inoculated fungal endophyte *A. cf. terreus* CJR-4 was confirmed by re-isolation from surface-sterilized root segments at 14 and 28 DAT (Fig. 14a, b). Gradual establishment of CJR-4 was observed inside the root tissues, as evidenced by colonization frequency increased from 30% at 14 DAT to 65% at 28 DAT (Fig. 14d). The re-isolated fungus showed colony morphology and microscopic characteristics identical to those of the original inoculated fungus (CJR-4), confirming its identity (Fig. 14b, c). Furthermore, microscopic examination of stained root tissues confirmed the presence of fungal hyphae, conidiophores, and conidia, providing additional evidence of successful root colonization by the fungal isolate CJR-4 (Fig. 14e, f).

## Discussion

Fungal endophytes promote plant growth by secreting phytohormones, enhancing mineral absorption, and protecting from phytopathogens. This potent plant growth-promoting fungus (PGPF) emerged as an alternative to chemical fertilizers. In this study, three distinct endophytic fungi were isolated from the roots of *Crotalaria juncea* L. plants and tested for their antagonistic, antioxidant, and plant growth-promoting potential.

The antagonistic potential of three isolated endophytic fungi was evaluated against two phytopathogenic fungi, *F. oxysporum* and *A. alternata*, using the dual culture method. The study demonstrated that CJR-4 showed potent mycelial inhibition against both tested pathogenic fungi, possibly due to the secretion of active biological compounds. An earlier study reported that endophytic fungi are a treasure trove of biologically active compounds with diverse effects, including antimicrobial, antioxidant, anticancer, insecticidal, antiviral, nematocidal, and plant growth-promoting activities^5,43^. Furthermore, the ethyl acetate crude extract of *A. cf. terreus* CJR-4 exhibited potent antifungal activity against both fungal pathogens tested. The observed antifungal activity may be attributed to the combined effect of diverse bioactive metabolites present in the fungal extract. A dose-dependent increase in antifungal activity was observed, with the crude extract showing greater sensitivity against *A. alternata* than *against F. oxysporum*, indicating differential effects of the bioactive metabolites present in the crude extract on the tested fungal pathogens (Fig. 4; Table 2). Similarly, antifungal activities of endophytic fungal crude metabolites have been reported against several phytopathogenic fungi^13,44,45^. The clear inhibition zone observed in the agar well diffusion assay further supports the role of diffusible bioactive secondary metabolites in pathogen inhibition. It corroborates the antagonistic activity observed during dual-culture test. This study reported the identification of diphenyl sulphone in the GC-MS chromatograms of ethyl acetate extract of *A. cf. terreus* CJR-4 (Fig. 5). Similarly, Sathyalingam et al.^46^ reported the detection of diphenyl sulphone in the GC-MS profile of the crude extract of an endophytic fungus *Fusarium avenaceum* isolated from leaf litter of *Elaeocarpus ganitrus*. Likewise, Jaffer et al.^47^ recently identified diphenyl sulphone in the crude extract of *Clonostachys rosea* CR 02, an entomopathogenic fungus active against *Myzus persicae*. Due to the presence of the -SO2- functional group, this compound exhibits excellent chemical and thermal stability, as well as antimicrobial, antioxidant, and enzyme-inhibitory activities^48,49^. Alhaddad et al.^13^ recently reported the presence of two potent compounds, hexadecanoic acid and lovastatin, in the dichloromethane extract of an endophytic fungal isolate, *Aspergillus terreus*. In contrast, lovastatin was not detected in the GC-MS analysis of the ethyl acetate crude extract of the fungus *A. cf. terreus* CJR-4 in the present study. Although *Aspergillus terreus* has been widely documented as an industrially important producer of lovastatin, secondary metabolite production in this species is highly strain-dependent and influenced by cultivation conditions and nutrient availability^50^^,^^51^. Therefore, the absence of lovastatin in CJR-4 likely reflects intraspecific metabolic variation rather than inconsistency with its taxonomic identity. Variation in metabolite profiles across studies may be linked to differences in fungal strains, solvent systems, and cultivation conditions. Furthermore, sulfur-containing fungal metabolites are common constituents of fungal secondary metabolism, which exhibit potent antimicrobial and antioxidant activities^52^. Other detected compounds, such as 2-nonen-1-ol (unsaturated alcohol), have been previously reported as common constituents of microbial and plant-derived extracts and exhibit antimicrobial and antioxidant activities by disrupting pathogen membranes and scavenging ROS^53,54^. Similarly, crude metabolites of the endophytic isolate CJR-4 exhibited excellent DPPH and ABTS^+^ radical scavenging activities. The presence of bioactive metabolites such as 1H-indene, 1-methylene (an aromatic hydrocarbon), and ethanone, 2-(formyloxy)−1-phenyl- (aromatic ketone derivatives) in the crude extract of CJR-4 might contribute to free radical scavenging activity, which is crucial for protecting against oxidative damage in the plant cells, as previously reported by El-Shamy et al.^55^ and Sasidharan et al.^56^.

Prior to the pot experiment, all three isolates were tested for their plant growth-promoting traits under *in vitro* conditions. The most common phytohormone, IAA, was tested, revealing that CJR-1 secreted the highest concentration, ranging from 222.49 ± 0.34 to 305.69 ± 0.41 μg/mL at 1 mg/mL to 5 mg/mL L-tryptophan concentrations. On the other hand, CJR-2 and CJR-4 exhibited maximum IAA production (104.05 ± 3.89 and 23.22 ± 0.52 µg/mL) at 2 mg/mL of L-tryptophan concentration. However, at a L-tryptophan concentration of 5 mg/mL, IAA production by isolates CJR-2 and CJR-4 gradually decreased, suggesting that the precursor concentration influenced IAA production (Fig. 6). The decline in IAA production by these two isolates at higher precursor concentration may be attributed to feedback regulation of IAA biosynthesis or diversion of L-tryptophan into alternative metabolic pathways. Similar trends of findings have also been reported in other fungal endophytes. For example, Numponsak et al.^57^ reported that fungal endophyte *Colletotrichum fructicola* CMU-A109 produced maximum IAA at an optimal L-tryptophan concentration (8 μg/mL), followed by a decline at higher precursor levels. Similarly, two fungal endophytes, *C*. *fructicola* CMU-AU 006 and *Tulasnella* sp. CMU-SLP 007 showed increased IAA production with increasing L-tryptophan concentration, but decreased after the precursor concentration reached 6 mg/mL^58^. Recently, Maan and Abdelhamid reported^59^ a similar observation: where the fungal endophyte *F. oxysporum* AUMC 16,438 produced the highest IAA (35.65 µg/mL) at 0.6% L-tryptophan concentration, but at higher concentrations (0.8–1.25%) negatively influenced IAA production. An earlier report showed that some endophytic fungi, such as *Aspergillus*, *Fusarium*, *Penicillium*, *Phoma*, *Piriformospora*, and *Chaetomium,* can produce IAA^8,60^. These IAA-producing endophytic fungi stimulate lateral root formation and root hair development, facilitating nutrient absorption in the host plant^61^. Solubilization of inorganic phosphate by microorganisms is mediated by organic acids and metabolites secreted during their metabolism, and this process is crucial when phosphate is a limiting factor. Phosphate-solubilizing strains have been reported to significantly enhance plant growth and crop yield by increasing phosphate uptake^62,63^. In this study, all tested endophytic fungi exhibited phosphate solubilization activity. Among the fungal isolates, CJR-2 showed the highest PSI of 3.09 ± 0.09, followed by CJR-4 and CJR-2 (Fig. 7). Similarly, endophytic fungi *Penicillium* sp. and *Aspergillus* sp. showed clear zones of P solubilization ranging from 1.0 to 1.2 mm^64^. Furthermore, all tested endophytes were capable of producing ammonia (Fig. 8), whereas one endophytic fungus, CJR-1, produced a siderophore. However, two isolates, CJR-2 and CJR-4, did not produce siderophores, possibly due to a lack of genes responsible for siderophore biosynthesis. Wang et al.^8^ reported that endophytic fungi *Fusarium* sp*.* ZZ13 and *Alternaria* sp. ZZ10 possesses multiple plant growth-promoting traits, including IAA, siderophore, and ammonia production, as well as phosphate solubilization. The endophytes *Aspergillus tubingensis* and *Talaromyces veerkampii* have also been reported to produce IAA and ammonia and to solubilize phosphate, thereby promoting overall rice plant growth^65^.

Maize is the world’s third-largest cereal crop after wheat and rice, and plays an important role in global agri-food systems. Due to increasing food consumption, global maize production has surged in the last few decades and is set to become the most cultivated and traded crop in the near future^66^. Hence, an approach has been adopted to evaluate the effects of isolated endophytes and AMF on the growth promotion of maize plants in pot conditions. Inoculating fungal endophytes into 10-day-old maize seedlings and observing for 28 days resulted in significant increases in plant height, biomass accumulation, and photosynthetic efficiency. Such enhancement may be due to PGPF’s functional role through mechanisms such as nutrient solubilization, nutrient availability, physiological responses, and modulation of the root system. Among the treatments, CJR-4 isolate exhibited the most pronounced effect, with significant increase in root length (30.33 ± 5.68 cm) and shoot length (86.36 ± 5.08 cm) as well as higher shoot fresh weight (45.93 ± 2.08 gm) and dry root weight (1.36 ± 0.03 gm) and dry shoot weight (8.57 ± 0.35 gm), might be due to their vigorous nutrient uptake and better physiological stimulation abilities making more prominent than other isolates (Table 10). It is noted that endophytic isolate CJR-4 inoculated maize plants surpass growth indices in comparison to AMF inoculated maize plants. Interestingly, the maximum root fresh weight observed under AMF treatment may be due to greater water accumulation, as mycorrhizal fungi are known to accumulate water. At the same time, the enhancement of root length by CJR-4 in maize plants may directly linked with colonization efficacy and root architecture development, which are pivotal for nutrient and water acquisition. This study confirmed the colonization of maize roots by the promising isolate *A. cf. terreus* CJR-4 through multiple validations, including re-isolation from surface-sterilized root segments, determination of colonization rate, identical morphology of the re-isolated fungus with the original isolate, and microscopic observation of stained root tissues. Colonization frequency increased from 30–65% at 14 and 28 DAT, indicating progressive root establishment of treated fungal endophyte. Successful colonization of fungal endophytes is crucial for plant-microbe interactions, facilitating nutrient mobilization, phytohormone production, and providing defense to their host through the secretion of diverse bioactive compounds. Therefore, the superior growth-promoting effects observed in CJR-4-inoculated maize plants may be attributed to its effective colonization potential and persistence within the root tissues. Similarly, Shan et al.^67^ reported that inoculation of the fungus *Paraphaosphaeria* sp. JRF11 efficiently colonized the tomato plant and boosted its growth. Likewise, Hu et al.^68^ found that early colonization of *Metarhizium* and *Pochonia* in hemp plants enhanced stem length and stem and root weight. Hence, effective colonization of fungal endophytes is crucial for improving plant growth and promoting plant health, irrespective of their multiple plant-growth-promoting traits^69^.

Photosynthetic pigments are crucial for increasing the photosynthetic rate in plants. In this study, inoculation with AMF and endophytic fungi resulted in the highest accumulation of photosynthetic pigments compared to uninoculated plants. Maize plant treated with AMF found to consist of a high amount of chlorophyll a (6.50 ± 0.07), chlorophyll b (1.39 ±0.05), chlorophyll a+b (7.89 ±0.13), and carotene (2.24 ± 0.04) in mg/gm fresh weight tissues (Table 11). Similarly, this finding is consistent with inoculation of AMF in maize plants, which has been reported to significantly increase chlorophyll a, chlorophyll b, and total chlorophyll (a+b) content, leading to enhanced photosynthetic rate and nutrient accumulation compared to untreated plants^70^. On the other hand, among the treatments, CJR-4 also resulted in substantial increases in chlorophyll a+b (6.94 ±0.10) and carotene (1.00 ±0.00) in mg/gm fresh weight tissue compared to the control (Table 11), suggesting their ability to enhance pigment biosynthesis. Likewise, inoculation with the endophytic fungus *Fusarium* sp. YMYD, *Alternaria* sp. ZZ10 and *Fusarium* sp. ZZ1 with tobacco plant reported to increase chlorophyll a, and total chlorophyll content^8^. Moreover, our findings are consistent with those of Jan et al.^71^, who reported that inoculation with the endophytic fungus *Candida membranifaciens* enhances chlorophyll and carotenoid levels in maize plants, making it a promising agent to boost photosynthetic pigments and overcome salt stress. Gul et al.^72^ reported that endophytic isolate *Aspergillus welwitschiae* BK enhanced the physiological performance of maize plants by inducing chlorophyll and carotenoid content under salinity stress. These overall results strongly corroborate the earlier report of Sun et al.^70^, which showed that AMF association with maize plants boosts chlorophyll accumulation, leading to increased photoprotection and enhanced physiological performance. An increasing level of carotene also showed its effect on photoprotective capabilities by minimizing the photooxidative damage and supporting photosystem II. Overall, the results demonstrated that our isolates (CJR-4) and AMF improved photosynthetic pigment accumulation in maize, likely through increased nutrient availability and metabolic regulation.

## Conclusion

This study demonstrated that fungal endophytes isolated from root tissues of *Crotalaria juncea* L. possess promising multifunctional activities, including antagonistic, antioxidant and plant growth-promoting potential. The present findings highlight the ability of selected fungal endophytes to enhance maize growth under pot conditions. Among the tested isolates, *A. cf. terreus* CJR-4 emerged as a promising isolate for further development as a bioinoculant. However, additional field studies are necessary to validate its long-term efficacy, ecological compatibility, and impact on soil microbial dynamics and health before large-scale agricultural use.

## Supplementary Information

Supplementary Information.

## Data Availability

The data supporting the findings of this study are available in the supplementary material of this manuscript.
