# Supplementary material for: Functional characterization of fungal endophytes with antagonistic and plant growth-promoting activities in maize
Source: Sci Rep. 2026 Jul 11;16:23638. doi: 10.1038/s41598-026-60768-4 (PMC13424326; doi:10.1038/s41598-026-60768-4)
Supplement: Supplementary file 1 — Supplementary Information. [file 41598_2026_60768_MOESM1_ESM.docx]

**
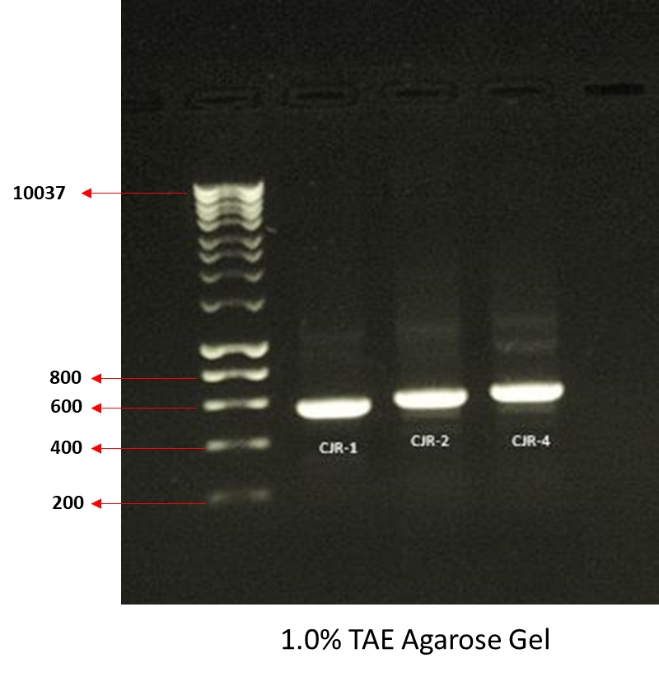
**

**Figure.**

Agarose gel electrophoresis of PCR-amplified internal transcribed spacer (ITS) region of endophytic fungal isolates using universal primers ITS1 and ITS4. Lane 1: DNA molecular weight marker (1000 bp ladder). Lanes CJR-1, CJR-2, and CJR-4 show single clear amplicons of approximately ~550–600 bp, confirming successful amplification of fungal ITS regions.

**FASTA SEQUENCE**

**>CJR1_ITS**

**TGCGGAGGGATCATTACCGACAGTCGACTAGCACTATCAGCCCGCTCCC**

**GGTAAAACGGGACGGCCCGCCAGAGGACCCCTAAACTCTGTTTCTATAT**

**GTAACTTCTGAGTAAAACCATAAATAAATCAAAACTTTCAACAACGGATC**

**TCTTGGTTCTGGCATCGATGAAGAACGCAGCAAAATGCGATAAGTAATG**

**TGAATTGCAGAATTCAGTGAATCATCGAATCTTTGAACGCACATTGCGCC**

**CGCCAGTATTCTGGCGGGCATGCCTGTTCGAGCGTCATTTCAACCCTCA**

**AGCACAGCTTGGTGTTGGGACTCGCGTTAATTCGCGTTCCTCAAATTGAT**

**TGGCGGTCACGTCGAGCTTCCATAGCGTAGTAGTAAAACCCTCGTTACT**

**GGTAATCGTCGCGGCCACGCCGTTACAACTTCTGAATGTTGACCTCGGA**

**TCAGGTAGGAATACCCGCTGAACTTAAGCATA**

**>CJR2_ITS**

**AAGGATCATTACTGAGTGAGGGCCCTCTGGGTCCAACCTCCCACCCGTG**

**TTTAACGAACCTTGTTGCTTCGGCGGGCCACGATCGTACCATCCGCCCC**

**CGGGCCCGCGCCCGCCGAAGACACCTGTGAACTCTGTCTGAAGTTGCAG**

**TCTGAGAAACTATTTAAATTAGTTAAAACTTTCAACAACGGATCTCTTGG**

**TTCCGGCATCGATGAAGAACGCAGCGAAATGCGATAAATAATGTGAATT**

**GCAGAATTCAGTGAATCATCGAGTCTTTGAACGCACATTGCGCCCTCTG**

**GTATTCCGGAGGGCATGCCTGTCCGAGCGTCATTGCTGCCCTCAAGCAC**

**GGCTTGTGTGTTGGGCCCCGTCCCCCCCCTCCCCGGGGGGACGGGCCC**

**GAAAGGCAGCGGCGGCACCGCGTCCGGTCCTCGAGCGTATGGGGCTTT**

**GTCACCCGCTCTTGTAGGCCCGGCCGGCGCTTGCCGACCCCCTCAATAC**

**AGGTTGACCTAGCTACCAGGTAGGGATACCCGCTGAACTTAAGCATATC**

**AATAA**

**>CJR4_ITS**

**TCGGACGATCATGGTCACCTCCCACCCGTGACTATTGTACCTTGTTGCTT**

**CGGCGGGCCCGCCAGCGTTGCTGGCCGCCGGGGGGCGACTCGCCCCCG**

**GGCCCGTGCCCGCCGGAGACCCCAACATGAACCCTGTTCTGAAAGCTTG**

**CAGTCTGAGTGTGATTCTTTGCAATCAGTTAAAACTTTCAACAATGGATC**

**TCTTGGTTCCGGCATCGATGAAGAACGCAGCGAAATGCGATAACTAATG**

**TGAATTGCAGAATTCAGTGAATCATCGAGTCTTTGAACGCACATTGCGCC**

**CCCTGGTATTCCGGGGGGCATGCCTGTCCGAGCGTCATTGCTGCCCTCA**

**AGCCCGGCTTGTGTGTTGGGCCCTCGTCCCCCGGCTCCCGGGGGACGG**

**GCCCGAAAGGCAGCGGCGGCACCGCGTCCGGTCCTCGAGCGTATGGGG**

**CTTCGTCTTCCGCTCCGTAGGCCCGGCCGGCGCCCGCCGACGCATACAG**

**CTACCAGGTTGACCTCGGATCAGGTAGGGATACCCGCTGAACTTAAGCA**

**TATCAATAAGACGGAGGAA**
